# Impact of COVID-19 on Pregnancy Outcomes across Trimesters in the United States

**DOI:** 10.3390/biomedicines11112886

**Published:** 2023-10-25

**Authors:** Shiza Virk, Karthik Gangu, Adeel Nasrullah, Aaisha Shah, Zohaa Faiz, Umair Khan, David Bradley Jackson, Anam Javed, Asif Farooq, Briana DiSilvio, Tariq Cheema, Abu Baker Sheikh

**Affiliations:** 1 Department of Internal Medicine, Allegheny Health Network, Pittsburgh, PA 15212, USA; shiza.virk@ahn.org (S.V.); aaishashah5@gmail.com (A.S.); anam.javed@ahn.org (A.J.); 2 Department of Internal Medicine, University of Kansas Medical Center, Kansas City, KS 66160, USA; kgangu2@kumc.edu; 3 Division of Pulmonology and Critical Care, Allegheny Health Network, Pittsburg, PA 15212, USA; adeel.nasrullah@ahn.org (A.N.); briana.disilvio@ahn.org (B.D.); tariq.cheema@ahn.org (T.C.); 4 Department of Medicine, School of Medicine, Aga Khan University, Karachi 74000, Pakistan; zohaa.faiz22@alumni.aku.edu; 5 Department of Internal Medicine, University of New Mexico, Albuquerque, NM 87106, USA; uakhan@salud.unm.edu (U.K.); dbjackson@salud.unm.edu (D.B.J.); 6 Department of Family and Community Medicine, Texas Tech Health Sciences Center, Lubbock, TX 79409, USA; afarooq76@gmail.com

**Keywords:** COVID-19, pregnancy, trimester-specific outcomes, mortality, racial disparities, complications, National Inpatient Sample, HELLP, preeclampsia, preterm birth

## Abstract

Background: Current knowledge regarding the association between trimester-specific changes during pregnancy and COVID-19 infection is limited. We utilized the National Inpatient Sample (NIS) database to investigate trimester-specific outcomes among hospitalized pregnant women diagnosed with COVID-19. Results: Out of 3,447,771 pregnant women identified, those with COVID-19 exhibited higher in-hospital mortality rates in their third trimester compared with those without the virus. Notably, rates of mechanical ventilation, acute kidney injury, renal replacement therapy, and perinatal complications (preeclampsia, HELLP syndrome, and preterm birth) were significantly elevated across all trimesters for COVID-19 patients. COVID-19 was found to be more prevalent among low-income, Hispanic pregnant women. Conclusions: Our findings suggest that COVID-19 during pregnancy is associated with increased risk of maternal mortality and complications, particularly in the third trimester. Furthermore, we observed significant racial and socioeconomic disparities in both COVID-19 prevalence and pregnancy outcomes. These findings emphasize the need for equitable healthcare strategies to improve care for diverse and socioeconomically marginalized groups, ultimately aiming to reduce adverse COVID-19-associated maternal and fetal outcomes.

## 1. Introduction

Pregnant women are a high-risk group for severe coronavirus disease (COVID-19) caused by severe acute respiratory syndrome coronavirus 2 (SARS-CoV-2) infection [1,2]. Prognostic factors for severe disease include advanced age, obesity, and comorbidities such as hypertension, diabetes, and coronary artery disease [3,4]. Of particular note, pregnancy is accompanied by respiratory and immune adaptations, primarily to benefit the growing fetus, which increase the risk of acquiring infections potentially leading to severe maternal–fetal complications [5]. The mechanisms that predispose pregnant patients to COVID-19 infection and disease complications are multifaceted. Chief among these are unique modifications in the maternal immune system in response to fetal tissue, changes in functional residual capacity, alterations in cardiovascular function, and variations in coagulation. These alterations are thought to heighten the risk of viral infections and certain bacterial and fungal infections [6,7,8,9,10]. Drawing on the experiences from the 2002 SARS-CoV-1 pandemic, which led to high mortality rates among pregnant women, it has become evident that pregnant individuals constitute a high-risk population for COVID-19 infection [11,12].

Several previous studies, including the large multinational cohort study known as the INTERCOVID trial, have shown that pregnant women with COVID-19 face a higher risk of maternal morbidity and mortality compared with a COVID-19 negative cohort [13]. However, our understanding of trimester-specific mortality and outcomes, which are discussed in our study, is still limited. Although multiple observational studies have highlighted COVID-19’s tendency to cause disproportionate mortality and morbidity in pregnant patients, early data did not indicate significant differences in outcomes [14,15,16]. At the onset of the pandemic, studies suggested only mild infections, slightly higher incidence, and no definitive evidence of worsened mortality, morbidity, or risk of vertical transmission [17,18]. Tosseta et al. showed that pregnancies affected by COVID-19 had higher incidence of preeclampsia compared with COVID-19-negative pregnancies [19]. However, these studies lacked high-quality data necessary to draw unbiased conclusions and failed to explore the relationship between the timing of COVID-19 infection and its effects on trimester-specific outcomes. Additionally, population-based estimates of COVID-19 diagnoses in pregnancy, segmented by race, ethnicity, and insurance status, are also limited.

In light of these gaps, our study aims to investigate the trimester-specific outcomes in pregnant patients after contracting COVID-19. This will enhance our understanding of the vulnerabilities of this high-risk population. Utilizing the large National Inpatient Sampling database, we measure outcomes such as in-hospital mortality and assess the impact of pre-existing comorbidities, social factors, financial constraints, and regional influences. Additionally, we examine trimester-specific maternal and fetal complications in the context of COVID-19 infection.

## 2. Materials and Methods

### 2.1. Data Source

The study was conducted using the US National Inpatient Sample (NIS) database from 2020. The NIS is the largest all-payer healthcare database in the United States, developed by the Agency of Healthcare Research and Quality Utilization Project [20]. The NIS contains data on approximately 20% stratified samples of all discharges from US hospitals, equivalent to 7 to 8 million hospital discharges per annum. This study involved the analysis of deidentified data and was exempt from institutional review board approval.

### 2.2. Study Design

In this retrospective case control study, we identified all the hospitalizations in the year 2020 with a primary or secondary diagnosis of pregnancy complicated by COVID-19 infection using the International Classification of Diseases (ICD), 10th Revision, Clinical Modification, ICD code. We excluded all hospitalizations with any missing information. There were a total of 1.6 million hospitalizations in 2020 with COVID-19, out of which 53,025 hospitalizations included a diagnosis of pregnancy.

### 2.3. Study Population

All the pregnant women who were 18 years of age or older admitted to the hospital with COVID-19 infection were included in the study. Briefly, International Classification of Diseases, 10th Revision, Clinical Modification (ICD-10-CM) diagnosis codes were used to retrieve patient samples with comorbid conditions, and ICD-10 procedure codes were used to identify inpatient procedures (Supplementary Table S1). The NIS database contains data regarding in-hospital outcomes, procedures, and other discharge-related information. Variables were divided into patient-related, hospital-related, and indicators of illness severity, as below:

- Patient: age, race (White, Black, Hispanic, Native American, Asian, other), insurance status (Medicare, Medicaid, private insurance, self-payment, no charge), median income based on patient’s zip code, and disposition.
- Hospital: location, teaching status, size in terms of number of beds, and region.
- Illness severity: length of stay (LOS), mortality, hospitalization cost, comorbidities, mechanical ventilation, circulatory support, and vasopressor use.
- The primary outcome was in-hospital mortality.
- Secondary outcomes included intubation and mechanical ventilation, vasopressor use, acute kidney injury (AKI), acute kidney injury requiring hemodialysis, venous thromboembolism.
- Trimester-based outcomes included molar pregnancy, ectopic pregnancy, missed abortion, threatened abortion, spontaneous abortion, gestational hypertension, gestational diabetes, preterm labor, eclampsia, preeclampsia, HELPP.
- Other outcomes included length of stay, the financial burden on healthcare, and resource utilization.

### 2.4. Variables and Statistical Analysis

All analysis was performed using Stata software version 17.0 (Stata Corporation, College Station, TX, USA). Sample weights provided by NIS were used to calculate national estimates. Descriptive statistics were used to summarize the continuous and categorical variables. Continuous variables were summarized as mean ± SD (standard deviation), categorical data as numbers and percentages. A two-tailed *p* value of ≤0.05 was considered statistically significant. For primary and secondary outcomes, univariate regression was used to calculate the unadjusted odds ratio for variables of interest, and *p* values of <0.2 from the univariate logistic regression were used to build a multivariate logistic regression model to adjust for potential confounders. The multivariate linear regression model was used for continuous variables (LOS and total hospital charge). The diagnoses and reasons for admission of the control group (COVID-19 negative) are detailed in the Supplementary Tables S2–S4.

## 3. Results

### 3.1. First Trimester

The first-trimester subgroup consisted of 1,160 pregnant COVID-19-positive patients. The mean age of the female patients was 29.3 years (SD: 6.28), with the most prevalent age group between 18 and 29 years (51.72%). There were significant differences in the racial composition of the two groups. COVID-19-positive individuals in the first trimester included a higher percentage of African Americans (34.5% vs. 30.8%, *p* < 0.001), Hispanics (33.62% vs. 21.46%, *p* < 0.001), and Asians or Pacific Islanders (4.7% vs. 2.9%, *p* < 0.001) compared with COVID-19-negative individuals. Conversely, COVID-19-negative individuals included a higher percentage of Caucasians (39.4% vs. 18.1%, *p* < 0.001) Most of both the COVID-19-positive and COVID-19-negative individuals in the first trimester were admitted to urban teaching hospitals. However, COVID-19-positive individuals were in a higher percentage in this category compared with COVID-19-negative individuals (82.75% vs. 76.94%, *p* < 0.001). The proportions of insurance types (Medicare, Medicaid, private, self-pay) and median household income were similar for COVID-19-positive and COVID-19-negative individuals, with no statistically significant differences (*p* = 0.71) and (*p* = 0.55).

The prevalence of various comorbidities differed between COVID-19-positive and COVID-19-negative individuals in the first trimester. Significant differences were observed for obesity (18.1% vs. 7.9%, *p* < 0.001). However, there were no statistically significant differences in the prevalence of diabetes (6.0% vs. 6.9%, *p* = 0.62), coronary artery disease (CAD) (0.4% vs. 0.3%, *p* = 0.83), alcohol abuse (1.3% vs. 1.6%, *p* = 0.70), hypertension (2.6% vs. 10.0% *p* = 0.01), smoking (13.8% vs. 21.4%, *p* = 0.004), drug abuse (7.8% vs. 14.3%, *p* = 0.005), chronic kidney disease (1.7% vs. 0.6%, *p* = 0.03), or collagen vascular disorders (1.3% vs. 0.7%, *p* = 0.22) between the two groups (Table 1).

### 3.2. Second Trimester

The second-trimester subgroup consisted of 3495 pregnant COVID-19-positive patients, out of a total of 98,375 individuals. The mean age of the female patients was 30 years (SD: 6.27), which was slightly higher than the COVID-19-negative individuals (mean age: 28.8 years, SD: 6.2) (*p* < 0.001). When looking at age groups, there were significant differences between COVID-19-positive and COVID-19-negative individuals in the second trimester. The majority of both groups fell into the age group of 18–29 years. However, COVID-19-positive individuals were in a slightly lower percentage in this age group compared with COVID-19-negative individuals (45.9% vs. 52.2%, *p* = 0.001). Racial composition also showed significant differences between the two groups. COVID-19-positive individuals in the second trimester included a higher percentage of Hispanics (44.2% vs. 21.21%, *p* < 0.001) and a lower percentage of Caucasians (20.3% vs. 40%, *p* < 0.001) compared with COVID-19-negative individuals. Other racial groups, such as African Americans, Asians or Pacific Islanders, Native Americans, and others, were also present in varying proportions between the two groups. The majority of both COVID-19-positive and COVID-19-negative individuals in the second trimester were admitted to urban teaching hospitals. However, COVID-19-positive individuals were in a slightly higher percentage in this category compared with COVID-19-negative individuals (84.7% vs. 83.2%, *p* = 0.004). COVID-19-positive individuals in the second trimester included a higher percentage with Medicaid coverage (56.57% vs. 53.62%, *p* = 0.008) and a lower percentage with private insurance (36% vs. 41.3%, *p* = 0.008) compared with COVID-19-negative individuals.

The prevalence of comorbidities also differed between COVID-19-positive and COVID-19-negative individuals in the second trimester. Significant differences were observed in the prevalence of smoking (8.9% vs. 17.9%, *p* < 0.001), drug abuse (3.1% vs. 9.2%, *p* < 0.001), and obesity (18% vs. 11.8%, *p* < 0.001). However, there were no statistically significant differences in the prevalence of hypertension (1.3% vs. 0.9%, *p* = 0.24), diabetes (5.4% vs. 4.6%, *p* = 0.29), coronary artery disease (CAD) (0.1% vs. 0.2%, *p* = 0.61), alcohol abuse (0.3% vs. 0.8%, *p* = 0.12), collagen vascular disorders (1.0% vs. 0.9%, *p* = 0.85), or chronic kidney disease (CKD) (0.7% vs. 0.6%, *p* = 0.75) between the two groups (Table 2).

### 3.3. Third Trimester

A total of 48,445 pregnant COVID-19 patients comprised the third-trimester subgroup. Their mean age was 28.21 years (SD: 6.02), with 57.3% between 18 and 29 years. The distribution of age groups differed significantly between the two groups. COVID-19-positive pregnant individuals were in a higher percentage in the age groups of 18–29 (57.3% vs. 50.0%, *p* < 0.001) and 30–39 (37.4% vs. 45.4% *p* < 0.001) compared with COVID-19-negative pregnant individuals. Conversely, COVID-19-negative pregnant individuals were in a higher percentage in the age group ≥40 (3.3% vs. 3.6% *p* < 0.001). There was a statistically significant difference in the racial distribution between the two groups. COVID-19-positive pregnant individuals included a higher percentage of Hispanics (43.1% vs. 21.2%, *p* < 0.001) and a lower percentage of Caucasians (28.6% vs. 52.4%, *p* < 0.001) compared with COVID-19-negative pregnant individuals. COVID-19-positive pregnant individuals showed a different distribution across median household income categories compared with COVID-19-negative pregnant individuals. Most patients in both cohorts fell into the USD <49,999 income category, with a higher percentage of COVID-19-positive pregnant individuals (35.2% vs. 27.5%, *p* < 0.001). There was a significant difference in the distribution of insurance status between the two groups. COVID-19-positive pregnant individuals included a higher percentage of those with Medicaid coverage (60.3% vs. 43.0%, *p* < 0.001) and a lower percentage with private insurance (34.9% vs. 54.0%, *p* < 0.001) compared with COVID-19-negative pregnant individuals. The distribution of hospital divisions, size in terms of number of beds, and hospital teaching status differed significantly between the two groups. Most of the patients in both cohorts were in large hospitals, with COVID-19-positive pregnant women representing a higher percentage (53.8% vs. 50.6%, *p* < 0.001). Most patients were admitted to urban teaching hospitals (80.9% vs. 74.2%, *p* < 0.001). More patients in both cohorts were admitted to South Atlantic hospitals (19.8% vs. 19.7%, *p* < 0.001) than others.

In the third-trimester pregnant population, significant differences were observed in certain comorbidities between those who tested positive for COVID-19 and those who tested negative. Pregnant women with diabetes were more prevalent in the COVID-19-positive group compared with the COVID-19-negative group (1.7% vs. 1.1%, *p* < 0.001). Conversely, a higher proportion of smokers were found in the COVID-19-negative group compared with the COVID-19-positive group (6.57% vs. 11.05%, *p* < 0.001). Additionally, pregnant women with a history of drug abuse were more common in the COVID-19-negative group compared with the COVID-positive group (2.0% vs. 3.1%, *p* < 0.001). Finally, obesity was significantly higher in the COVID-19-positive group compared with the COVID-19-negative group (13.8% vs. 11.2%, *p* < 0.001). However, there were no statistically significant differences in the prevalence of hypertension (0.08% vs. 0.09%, *p* = 0.93), coronary artery disease (CAD) (0.05% vs. 0.04%, *p* = 0.86), alcohol abuse (0.06% vs. 0.09%, *p* = 0.25), collagen vascular disorders (0.4% vs. 0.4%, *p* = 0.71), or chronic kidney disease (CKD) (0.06% vs. 0.11%, *p* = 0.18) between the two groups (Table 3).

The COVID-19-positive cohort had a higher proportion of obesity in all three trimesters compared with the non-COVID-19 cohort (18.1% vs. 7.9%, double in the first trimester, 18.02% vs. 11.1% in the second trimester, 13.8% vs. 11.1% in the third trimester), while chronic kidney disease (CKD) rates were slightly higher only in the first trimester (1.7% vs. 0.6%, *p* < 0.03) and diabetes in the third (1.7% vs. 1.1%, *p* < 0.001). Smoking (21.4% in COVID-19-negative vs. 13.8% in COVID-19-positive in the first followed by 17.9% vs. 8.9% in the second and 11.1% vs. 6.6% in the third trimester), drug use (14.3% vs. 7.8% in the first, 9.2% vs. 3.1% in the second, and 3.1% vs. 2.03% in the third trimester), and hypertension (HTN) (10% vs. 2.6% only in the first trimester) were significantly higher in non-COVID-19 pregnant women compared with COVID-19-positive women.

No significant difference between COVID-19-positive and negative pregnant patients was found in terms of median household income or hospital division status in the first and second trimesters, and insurance status was not significant in the first trimester. No significant differences in comorbidities like coronary artery disease (CAD), alcohol use, or collagen vascular disease were found between the two groups across any of the three trimesters.

### 3.4. In-Hospital Outcomes

In-hospital outcomes were further characterized into overall mortality, adverse outcomes, and in-hospital quality metrics including LOS, financial costs, and follow-up disposition.

### 3.5. In-Hospital Mortality

Pregnant patients who tested positive for COVID-19 had higher rates of mortality of about 0.43% in the first trimester, 0.28% in the second trimester, and 0.09% in the third trimester. Subgroup comparative analysis performed based on each individual trimester showed mortality was only a significant factor in the third trimester (0.09% vs. 0.004%, aOR 24.4; 95% CI 10.7–55.63, *p* < 0.001) compared with the first (*p* < 0.08) and second trimesters (*p* < 0.20) for COVID-19-positive pregnant women compared with COVID-19-negative pregnant women.

### 3.6. In-Hospital Complications

#### 3.6.1. First Trimester

Patients who presented with COVID-19 during the first trimester required more mechanical ventilation (2.2% vs. 0.7%, aOR: 3.3 [95% CI 1.3–8.4], *p* < 0.001) compared with those who presented without COVID-19.

However, this cohort did not experience higher rates of AKI (2.2% vs. 1.8%, aOR: 0.5 [95% CI 0.2–1.8], *p* = 0.29), venous thromboembolism (3.9% vs. 2.2%, aOR: 1.2 [95% CI 0.6–2.56], *p* = 0.64), threatened abortion (0.9% vs. 1.3%, aOR: 0.6 [95% CI 0.2–2.5], *p* = 0.51), missed abortion (2.6% vs. 2.0%, aOR: 1.2 [95% CI 0.5–2.7], *p* = 0.67), or spontaneous abortion (4.3% vs. 3.6%, aOR: 0.98 [95% CI 0.50–1.9], *p* = 0.96).

This group of patients also did not experience an increased incidence of gestational hypertension (1.3% vs. 1.5%, aOR: 0.9 [95% CI 0.3–3.1], *p* = 0.91), gestational diabetes mellitus (3.0% vs. 1.9%, aOR: 1.3 [95% CI 0.6–2.9], *p* = 0.56), molar pregnancy (0.4% vs. 0.1%, aOR: 1.4 [95% CI 0.4–5.0], *p* = 0.58), or ectopic pregnancy (4.3% vs. 5.9%, aOR: 0.7 [95% CI 0.3–1.3], *p* = 0.23) (Table 4).

#### 3.6.2. Second Trimester

Women who presented with COVID-19 during the second trimester required more mechanical ventilation (4.2% vs. 0.6%, aOR: 5.6 [95% CI 3.5–9.0], *p* < 0.001). However, there was no significant difference in the presence of AKI (2.2% vs. 1.8%, aOR: 1.8 [95% CI 1.04–3.0], *p* = 0.03), AKI requiring hemodialysis (0.4% vs. 0.1%, aOR: 4.6 [95% CI 1.1–18.6], *p* = 0.03), or venous thromboembolism (1.1% vs. 0.6%, aOR: 1.6 [95% CI 0.8–3.3], *p* = 0.21).

There was also no significant difference in the incidence of threatened abortion (0.3% vs. 0.4%, aOR: 0.7 [95% CI 0.2–3.0], *p* = 0.62), spontaneous abortion (1.7% vs. 2.6%, aOR:0.6 [95% CI 0.3–1.1], *p* = 0.11), preterm labor (3.4% vs. 4.9%, aOR: 0.8 [95% CI 0.5–1.2], *p* = 0.30), HELLP syndrome (0.7% vs. 0.8%, aOR: 0.7 [95% CI 0.3–1.7], *p* = 0.41), gestational hypertension (1.6% vs. 2.2%, aOR: 0.8 [95% CI 0.4–1.4], *p* = 0.35), or gestational diabetes mellitus (6.9% vs. 3.7%, aOR: 1.4 [95% CI 1.01–1.9], *p* = 0.04). Interestingly, the cohort of patients who were COVID-19-negative had an increased risk of developing preeclampsia (2.9% vs. 6.1%, aOR: 0.4 [95% CI 0.2–0.61], *p* < 0.001) and having a missed abortion (2.0% vs. 5.9%, aOR: 0.35 [95% CI 0.2–0.6], *p* < 0.001) (Table 5).

#### 3.6.3. Third Trimester

Women who presented with COVID-19 during the third trimester had higher chances of presenting with preeclampsia (9.0% vs. 7.1%, aOR: 1.17 [95% CI 1.1–1.3], *p* < 0.001) and HELLP syndrome (0.5% vs. 0.2%, aOR: 1.78 [95% CI 1.30–2.4], *p* < 0.001). This cohort of women also required more mechanical ventilation (0.8% vs. 0.1%, aOR: 14.8 [95% CI 11.1–19.8], *p* < 0.001) and were more likely to develop AKI requiring hemodialysis (0.03% vs. 0.002%, aOR: 14.3 [95% CI 3.69–55.7], *p* < 0.001). However, there was no significant difference in the rate of development of gestational diabetes mellitus (9.9% vs. 9.2%; aOR 0.98 (95% CI 0.9–1.05), *p* = 0.68) or gestational hypertension (6.3% vs. 7.4%; aOR 0.9 (95% CI 0.8–0.96), *p* = 0.005). They also had no significant differences in the rates of venous thromboembolism (0.09% vs. 0.06%; aOR 1.3 (95% CI 0.7–2.6) *p* = 0.41), acute kidney injury (0.3% vs. 0.1%; aOR 1.7 (95% CI 1.2–2.5) *p* = 0.004), eclampsia (0.1% vs. 0.06%; aOR 1.6 (95% CI 0.9–2.7) *p* = 0.11), and preterm labor (1.2% vs. 0.7%; aOR 1.3 (95% CI 1.1–1.6) *p* = 0.01) (Table 6).

#### 3.6.4. In-Hospital Quality Measures and Disposition

More than 90% of the affected patients were discharged to home, followed by ~2–4% to home with home health, and 0.8–3.5% who left against medical advice (AMA). A small percentage were discharged to skilled nursing facilities, long-term acute care, or nursing homes (0.07–1.3%). Mean hospital charges were higher for the COVID-19-positive group in the latter two trimesters (i.e., adjusted total charge USD 16,000 in the second and USD 4000 in the third trimester, *p* < 0.001).

#### 3.6.5. First Trimester

More than 90% of the affected patients were discharged to home, followed by ~2–3% to home with home health, and 3.5% who left against medical advice (AMA). There was no significant difference between the two cohorts. The mean LOS (4.25 days vs. 2.88 days; adjusted length of stay: 1.24 days longer, *p* = 0.18) and mean total charge (USD 39,826 vs. USD 30,463; adjusted total charge: USD 2254, *p* = 0.60) were higher for the cohort of women who tested positive for COVID-19; however, these differences were not statistically significant.

#### 3.6.6. Second Trimester

More patients who tested positive for COVID-19 were discharged to home with home health (3.61% vs. 1.32%, *p* < 0.001). Most of the patients in both cohorts were discharged to routine care (92.76% vs. 94.25% *p* < 0.001). The mean total hospitalization charge for the cohort of women who presented with COVID-19 was significantly higher than for women who were negative for COVID-19 (USD 57,401 vs. USD 37,436; adjusted total charge: USD 16,213 higher, *p* < 0.001). Mean length of stay was not statistically significant between the two groups (5.02 days vs. 4.3 days; adjusted length of stay: 0.35 days longer, *p* = 0.2).

#### 3.6.7. Third Trimester

Most patients in both cohorts were discharged to routine care (97.88% vs. 98.83% *p* < 0.001). The mean total hospitalization charge for the cohort of women who presented with COVID-19 in the third trimester was significantly higher than for women who were negative for COVID-19 (USD 29,128 vs. USD 23,039; adjusted total charge: USD 4454 higher, *p* < 0.001). Mean length of stay was also longer for this cohort of women (2.78 days vs. 2.47 days; adjusted length of stay: 0.27 days longer, *p* < 0.001).

### 3.7. Obstetric Outcomes

Examining obstetric outcomes based on COVID-19 status, the second trimester data showed no significant difference in vaginal delivery and C-section rates between COVID-19-positive and COVID-19-negative groups (*p* values of 0.53 and 0.79, respectively).

Similarly, in the third trimester, while there appeared to be a slight variance in outcomes, the differences were not statistically significant at the threshold set at *p* < 0.001 (*p* values of 0.02 for vaginal delivery and 0.005 for C-section). Hence, COVID-19 status in these trimesters did not significantly impact the observed obstetric outcomes (Table 7 and Table 8).

## 4. Discussion

Our comprehensive, nationally representative investigation of pregnant women in the United States yielded several significant findings. We found these observations to vary according to each trimester of pregnancy. Notably, we observed a higher mortality rate in third-trimester patients diagnosed with COVID-19. Moreover, the incidence of AKI necessitating hemodialysis was significantly higher in the second and third trimesters among those with COVID-19. COVID-19-positive women demonstrated a heightened prevalence of preeclampsia and HELLP syndrome in the third trimester. This is in contrast with the COVID-19 negative cohort, which showed higher rates of these conditions in the second trimester. Furthermore, an elevated frequency of preterm labor was evident among COVID-19-positive pregnant women in their third trimester. In terms of demographic trends, we found that the highest occurrence of COVID-19 was recorded among pregnant Hispanic women, compared with other racial and ethnic groups. This trend was particularly pronounced in women with pre-existing obesity, those from low-income households, and women being treated in large urban teaching hospitals.

### 4.1. Mortality

Approximately 5% of women of reproductive age are pregnant at any given time in the general population [21]. Our research scrutinized 53,025 confirmed COVID-19-positive pregnancies across the United States, spanning from March to December of 2020. Preliminary findings showed seemingly comparable mortality rates across the first, second, and third trimesters of pregnancy. However, a deeper subgroup analysis uncovered a notable spike in mortality among third-trimester pregnant women with COVID-19. Additionally, as the pregnancies progressed through each trimester, we observed a progressively higher incidence of respiratory failure requiring mechanical ventilation compared with those unaffected by COVID-19. In line with our findings, a meta-analysis by Lassi et al. indicated that most women (77.7%) affected with COVID-19 were in their third trimester [22]. This trend was further supported by the COVID-NET report from the US Department of Health and CDC, which suggested that the majority of COVID-19-positive pregnant women admitted to US hospitals (87.4%) were in their third trimester, either for delivery or due to obstetric complications [23]. Our data underscore that pregnant women in their third trimester face higher rates of preeclampsia and HELLP syndrome, which may contribute to the observed increase in morbidity and, subsequently, mortality. Echoing this, a Lancet-published meta-analysis revealed that many leading causes of maternal death, which primarily occur in the third trimester and peripartum period, are hypertensive disorders, including gestational hypertension, preeclampsia, HELLP syndrome, and adverse cardiovascular outcomes [24].

### 4.2. Preeclampsia and HELLP

COVID-19’s impact on pregnant women has notably increased the prevalence of preeclampsia among this group [25]. Both COVID-19 and preeclampsia have a shared pathological underpinning: ‘endothelial damage’. Our study discovered that 9% of patients in their third trimester developed preeclampsia, and a further 0.5% developed HELLP syndrome. The multinational INTERCOVID study, which focused on the relationship between preeclampsia and COVID-19, found a strong and independent association between these conditions. Their findings revealed that both COVID-19 and preeclampsia independently contribute to preterm birth, severe perinatal morbidity, and mortality [25,26]. Moreover, the study suggests a potential cause–effect relationship between COVID-19 and preeclampsia, which appears to be independent of confounding factors [25,26]. Hence, markers for HELLP syndrome and preeclampsia in pregnant women, particularly those testing positive for COVID-19, warrant meticulous evaluation. Neglecting to identify these devastating feto–maternal outcomes could result in severe complications associated with preeclampsia if left untreated. Therefore, our study suggests considering COVID-19 as a possible risk factor for preeclampsia or HELLP syndrome. This correlation could explain the increased mortality rates observed among pregnant women in their last trimester and postpartum period. Our observations present a dichotomy in the relationship between COVID-19 and preeclampsia onset across trimesters. The decreased risk of preeclampsia in the second trimester among COVID-19 positive-patients deviates from the current literature, underscoring the possibility of varied physiological responses to the virus at different stages of pregnancy or the influence of external confounding factors. These could range from differences in patient demographics, viral strains, the retrospective nature of our study, and the limitations inherent to ICD-10 documentation or medical interventions. Conversely, the elevated risk noted in the third trimester aligns with existing research, reaffirming the understanding that COVID-19 might exacerbate conditions like preeclampsia through mechanisms like endothelial dysfunction or heightened inflammatory response. This study accentuates the need for continuous and nuanced exploration of the multifaceted impacts of COVID-19 on obstetric outcomes.

### 4.3. Comorbidities

Although most pregnant women with a COVID-19 infection are asymptomatic or display only mild symptoms, a small percentage may develop severe acute respiratory distress syndrome (ARDS) and multi-organ failure. Our study revealed that the risk of such severe outcomes increases significantly among those with pre-existing comorbidities such as diabetes, hypertension, smoking habits, and notably obesity. A case–control study by Vouga et al. identified pulmonary comorbidities, hypertensive disorders, and diabetes as significant risk factors for severe COVID-19 infection [27]. As these women progress towards severe or critical stages of the disease, the escalated effects of a hyperinflammatory state, multi-organ dysfunction, and relative hypoxemia can adversely impact the placenta and the developing fetus. Among the most prevalent comorbidities noted in our study was obesity, affecting nearly 18% of pregnant women within the first 26 weeks and 14% in the final trimester. Multiple studies have identified higher BMI as an independent marker for increased morbidity and mortality, which can result in poorer outcomes in pregnant women [28,29,30]. When this high BMI is accompanied by gestational weight gain, the compounded effects of obesity on health outcomes are apparent and are currently being investigated in several studies. For example, a large retrospective study conducted in France identified obesity as a significant factor associated with severe COVID-19 infection [31]. Therefore, it is crucial to understand these risk factors, particularly in groups like pregnant women who are known to be predisposed to complications. Armed with this knowledge, healthcare providers can better prepare and communicate effective preventive measures, such as vaccination, thus improving the provision of healthcare services.

### 4.4. Acute Kidney Injury

Our study identified a notable increase in the rates of acute kidney injury (AKI) and AKI requiring renal replacement therapy (RRT) among pregnant women in their second and third trimesters. The incidence of AKI has been observed in approximately 20–40% of COVID-19 patients, a significant proportion of whom require RRT [32,33]. Pregnant women, compared with their non-pregnant counterparts, exhibit a 51% increased risk of AKI, regardless of existing co-morbidities [34]. Given the increased likelihood of multi-organ failure and reliance on vasopressors, it is vital to acknowledge that acute respiratory distress syndrome (ARDS) can significantly affect renal function, as demonstrated in our study cohort. However, the etiology of kidney damage in pregnant women often involves multiple factors, including direct viral damage from SARS-CoV-2, which is mediated by viral replication after attachment to angiotensin-converting enzyme 2 receptors on host cells—these are plentiful in maternal kidneys, placenta, and the uterus during pregnancy [35]. This can potentially heighten the risk of mitochondrial dysfunction, acute tubular necrosis, and collapsing glomerulopathy [36,37].

### 4.5. Preterm Labor

Our study underscores that the rates of preterm labor are nearly 30% higher in pregnant women testing positive for COVID-19 in their third trimester, compared with the COVID-19 negative cohort. This finding aligns with an extensive international study demonstrating an escalating risk of preterm delivery beyond the 20-week gestational mark [38]. Additionally, a large retrospective study in California showed an increased risk of preterm birth and early term birth [39]. The heightened risk of preterm birth in the context of COVID-19 was also linked to co-existing medical conditions such as hypertension, diabetes, and obesity—as in our findings. Nonetheless, like other studies, our research has not conclusively established a cause–effect relationship between COVID-19 infection and preterm birth, highlighting the need for further prospective studies.

### 4.6. Obstretic Outcomes

Our study showed no significant difference in mode of delivery between COVID-19-positive and negative patients in the second or third trimester. This is in accordance with a retrospective, multicenter case–control study that showed COVID-19 during pregnancy does not increase the rate of Cesarean delivery [40]. This may be due to the fact our study controlled for other factors that may impact mode of delivery.

### 4.7. Racial, Financial, and Social Aspects of COVID-19 and Pregnancy

It is a well-established fact that COVID-19 in the setting of pregnancy is associated with poorer maternal and neonatal outcomes. Some of the adverse events include hospitalization, ICU stays, the need for ventilation, and higher rates of stillbirth and early delivery. Additionally, there are concerns about placental injury, the emergence of conditions resembling preeclampsia, increased maternal stress and depression, neurological issues in both mother and newborn, and the long-term effects of COVID-19 [41]. In our study, we discovered that individuals within the 18–39-years age bracket were most susceptible to COVID-19 infection, accounting for 40–59% of our study population, with a mean age of 28–29 years. This is congruent with data from the Centers for Disease Control and Prevention, which indicate a similar prevalence of COVID-19 within this age range in the larger pregnant demographic [42]. Upon dissecting our data by ethnicity, a distinct pattern emerged; Hispanic women consistently represented the highest percentage of COVID-19 infections throughout all trimesters, with an incidence ranging between 34 and 44%. Remarkably, African American women exhibited the second highest rates of infection, particularly prominent during the first trimester. These observations align with a comprehensive study conducted within a large Californian healthcare system encompassing 17,446 women, where it was reported that Hispanic women were 2.6 times more likely to contract COVID-19 compared with their White counterparts [43]. Similarly, research conducted in New York City corroborated these findings, demonstrating an elevated proportion of COVID-19 infection among pregnant Hispanic women [44]. A potential explanation for the higher infection rates observed within the Hispanic population may be cultural factors, embodied by the health belief model, as well as socioeconomic circumstances that potentially increase their exposure to the broader population, thereby heightening their susceptibility to infection.

Correspondingly, our study revealed that a significant portion of African American women were affected by COVID-19. A retrospective analysis conducted at the University Hospital of Cleveland Medical Center examined the social determinants of health among pregnant women from diverse racial and ethnic backgrounds [45]. That study indicated that African American women were more likely to experience occupational exposure and hail from areas of low income. Additionally, these women reported a higher prevalence of preterm deliveries, underlining the correlation between socioeconomic disparity and adverse pregnancy outcomes [45]. In Michigan, a study involving 1131 females demonstrated that African American women had nearly twice the risk of contracting COVID-19 compared with their White counterparts, even after adjustments for comorbidities [46]. These findings underscore the urgent need to explore the influence of COVID-19 on pregnancy among groups who, because of structural racism, often face inequitable birth outcomes.

While the data suggest that socioeconomic barriers pose significant challenges to the African American population, further examination of statistics from the National Immunization Survey indicates that African American women have the lowest rates of complete COVID-19 vaccination compared with their White and Hispanic counterparts [47]. A combination of clinical concerns, such as apprehension about potential adverse reactions, death, or disease resulting from the vaccine, and access barriers, including perceived difficulty obtaining the vaccine, shortages, and presumed vaccine costs, contribute to this discrepancy. These data highlight the necessity of additional efforts to reduce barriers to vaccination and improve health equity [48].

With respect to racial disparities, our study discerned that households with incomes in the lowest quartile (under USD 49,999) demonstrated the highest prevalence of COVID-19 among pregnant women, coupled with a high frequency of Medicaid insurance coverage. As household income increased, these rates exhibited a downward trend. Several factors, intricately tied to low socioeconomic status (SES), amplify the exposure and infection rates within this demographic. Housing conditions, often overcrowded due to financial constraints, are a substantial factor. As of 2020, the poverty rate in the United States stood at 11.4%, with African American and Hispanic populations registering the highest rates, at 19.5% and 17% respectively [49]. This economic reality means that these communities are more likely to live in crowded conditions, which inherently disrupts the ability to effectively follow social distancing guidelines [49]. Furthermore, individuals from low-SES groups often engage in sectors such as supermarkets and public transportation, where remote work is impractical. This lack of remote work opportunities exposes them to a heightened risk of infection [50]. Moreover, the precarious nature of their employment and income, particularly during the upheaval caused by the COVID-19 pandemic, exacerbates their vulnerability and underscores the intricate relationship between socioeconomic factors and health outcomes. To address racial and financial disparities in COVID-19 infection and vaccination intent among pregnant women during novel pandemics, future research and interventions should adopt a lens of financial health equity and identify strategies rooted in institutional trustworthiness and systems perspectives.

### 4.8. Limitations

The scope of our analysis is constrained by the lack of data on crucial aspects such as the timing of COVID-19 diagnosis during pregnancy, the severity of the disease, the variant of COVID-19 involved, and the vaccination status of the pregnant women—all of which can substantially influence the outcomes, including mortality rates [51,52,53]. Another significant gap is the absence of information on the effects of COVID-19-specific medication in the pregnant population and potential fetal issues, which is essential to assess the possibility of vertical transmission. Another significant limitation of our study is the database’s lack of data regarding the severity of COVID-19 infection, preventing stratified analyses that could offer deeper insights into the relationship between disease severity and preeclampsia onset. Furthermore, our data are confined to index hospitalization, offering little insight into the short- and long-term repercussions post-discharge. The use of ICD-10 codes also brings potential challenges, being prone to inaccuracies in coding or other errors that could lead to bias. Finally, despite meticulous efforts to mitigate confounding variables using multivariate analysis, the presence of residual confounding variables affecting the outcomes cannot be entirely ruled out.

### 4.9. Future Directions

Despite these limitations, our study helps to identify several areas that warrant further investigation to supplement the existing body of literature. A more detailed analysis of the timing and severity of COVID-19 during pregnancy, the variants involved, and the vaccination status of the patients would allow more nuanced understanding of disease progression and outcomes. Likewise, the impact of COVID-19-specific treatments on pregnant individuals, potential fetal implications, and possible vertical transmission needs extensive exploration. The collection and analysis of post-discharge data could provide valuable insights into the short- and long-term effects of COVID-19 on pregnant women and their children. Furthermore, continuous efforts to refine the coding practices and minimize errors can contribute to the validity of future studies.

## 5. Conclusions

Our analysis underscores the nuanced impact of COVID-19 on pregnant women, revealing outcomes that vary across trimesters and demographic groups. While trimester-specific outcomes offer critical insights into the clinical progression of COVID-19 during pregnancy, the observed disparities further emphasize the importance of an equitable and personalized care approach. Factors such as race and socio-economic status emerged as significant in our study. However, it is essential to understand these within a broader healthcare context. The susceptibility of pregnant women to COVID-19 complications necessitates an inclusive and equitable medical strategy, encompassing both clinical treatments and targeted preventive measures. Furthermore, the disparities observed serve as a reminder of the importance of context-driven, equitable healthcare. Care should be approached holistically, acknowledging not only the medical but also the environmental and social determinants influencing health outcomes. In navigating the challenges of this pandemic, our findings advocate for comprehensive, personalized, and data-driven healthcare protocols, promoting equitable access and outcomes for all. Embracing such a multifaceted approach is paramount to safeguarding both maternal and fetal health, especially as the pandemic landscape continues to evolve.

## Figures and Tables

**Table 1:** First-trimester pregnant patient-level characteristics.

Characteristics	COVID-19-Positive First Trimester (0 to 13 Weeks)	COVID-19-Negative First Trimester (0 to 13 Weeks)	*p* Value
**N = 39,695**	**1160**	**38,535**	
Mean Age in Years (SD)	29.3 (6.28)	28.1 (6.1)	0.01
Age Groups			0.11
<18	2.15%	1.79%	
≥18–29	51.72%	58.14%	
30–39	40.08%	36.38%	
≥40	6.03%	3.68%	
Race			<0.001
Caucasian	**18.1%**	**39.43%**	
African American	**34.48%**	**30.87%**	
Hispanic	**33.62%**	**21.46%**	
Asian or Pacific Islander	**4.74%**	**2.93%**	
Native American	**1.72%**	**0.84%**	
Other	**7.32%**	**4.46%**	
Median Household Income			0.55
USD <49,999	38.09%	38.72%	
USD 50,000–64,999	30.30%	27.51%	
USD 65,000–85,999	20.77%	20.03%	
USD >86,000	10.82%	13.74%	
Insurance Status			0.71
Medicare	2.24%	2.33%	
Medicaid	60.53%	56.67%	
Private	31.39%	34.49%	
Self-pay	5.82%	6.51%	
Hospital Division			0.10
New England	3.87%	3.15%	
Middle Atlantic	11.2%	13.08%	
East North Central	12.49%	13.23%	
West North Central	3.87%	4.9%	
South Atlantic	23.27%	22.75%	
East South Central	3.01%	6.86%	
West South Central	15.08%	15.86%	
Mountain	7.32%	6.8%	
Pacific	19.82%	13.36%	
Hospital Size			0.05
Small	15.08%	19.13%	
Medium	25.43%	29.53%	
Large	59.48%	51.34%	
Hosptal Teaching Status			<0.001
Rural	**0.86%**	**7.56%**	
Urban non-teaching	**16.37%**	**15.49%**	
Urban teaching	**82.75%**	**76.94%**	
Comorbidities			
Hypertension	2.58%	10%	0.01
Diabetes	6.03%	6.86%	0.62
Coronary artery disease	0.43%	0.35%	0.83
Smoking	13.79%	21.41%	0.004
Alcohol misuse	1.29%	1.61%	0.70
Drug misuse	7.75%	14.26%	0.005
Obesity	**18.1%**	**7.9%**	<0.001
Collagen vascular disorders	1.29%	0.65%	0.22
Chronic kidney disease	1.72%	0.61%	0.03

**Table 2:** Second-trimester pregnant patient-level characteristics.

Characteristics	COVID-19-Positive Second Trimester (14 to 26 Weeks)	COVID-19-Negative Second Trimester (14 to 26 Weeks)	*p* Value
**N = 98,375**	**3495**	**94,880**	
Mean age in years (SD)	29.99 (6.27)	28.8 (6.2)	<0.001
Age Groups			0.001
<18	1.71%	1.79%	
≥18–29	45.92%	52.23%	
30–39	45.77%	41.68%	
≥40	6.58%	4.29%	
Race			<0.001
Caucasian	**20.31%**	**40.04%**	
African American	**23.74%**	**29.42%**	
Hispanic	**44.2%**	**21.21%**	
Asian or Pacific Islander	**3.57%**	**3.71%**	
Native American	**1.43%**	**1.11%**	
Other	**6.72%**	**4.52%**	
Median Household Income			0.83
USD <49,999	37.41%	36.27%	
USD 50,000–64,999	27.05%	27.34%	
USD 65,000–85,999	21.29%	20.98%	
USD >86,000	14.24%	15.41%	
Insurance Status			0.008
Medicare	2.21%	1.89%	
Medicaid	56.57%	53.62%	
Private	36.04%	41.29%	
Self-pay	5.16%	3.2%	
Hospital Division			0.16
New England	2.71%	3.25%	
Middle Atlantic	13.3%	11.1%	
East North Central	11.15%	13.29%	
West North Central	4%	5.67%	
South Atlantic	20.88%	22.15%	
East South Central	6.72%	7.27%	
West South Central	17.45%	15.81%	
Mountain	6.58%	6.77%	
Pacific	17.16%	14.69%	
Hospital Size			0.27
Small	13.01%	13.83%	
Medium	28.04%	25.24%	
Large	58.94%	60.93%	
Hosptal Teaching Status			0.004
Rural	2.28%	5.03%	
Urban non-teaching	13.01%	11.81%	
Urban teaching	84.69%	83.15%	
Comorbidities			
Hypertension	1.28%	0.87%	0.24
Diabetes	5.43%	4.58%	0.29
Coronary artery disease	0.14%	0.24%	0.61
Smoking	**8.86%**	**17.87%**	<0.001
Alcohol misuse	0.28%	0.81%	0.12
Drug misuse	**3.14%**	**9.22%**	<0.001
Obesity	**18.02%**	**11.78%**	<0.001
Collagen vascular disorders	1%	0.93%	0.85
Chronic kidney disease	0.71%	0.62%	0.75

**Table 3:** Third-trimester pregnant patient-level characteristics.

Characteristics	COVID-19-Positive Third Trimester (27 Weeks to Birth)	COVID-19-Negative Third Trimester (27 Weeks to Birth)	*p* Value
**N = 3,363,831**	**48,445**	**3,315,386**	
Mean age years (SD)	**28.21 (6.02)**	**29.22 (5.8)**	<0.001
Age Groups			<0.001
<18	**1.95%**	**1.11%**	
≥18–29	**57.29%**	**49.98%**	
30–39	**37.48%**	**45.36%**	
≥40	**3.27%**	**3.55%**	
Race			<0.001
Caucasian	**28.6%**	**52.45%**	
African American	**17.28%**	**15.2%**	
Hispanic	**43.13%**	**21.21%**	
Asian or Pacific Islander	**3.66%**	**6.03%**	
Native American	**0.71%**	**0.75%**	
Others	**6.59%**	**4.37%**	
Median Household Income			<0.001
USD <49,999	**35.19%**	**27.47%**	
USD 50,000–64,999	**27%**	**26.1%**	
USD 65,000–85,999	**23.48%**	**24.1%**	
USD >86,000	**14.31%**	**22.33%**	
Insurance Status			<0.001
Medicare	**0.77%**	**0.64%**	
Medicaid	**60.34%**	**42.98%**	
Private	**34.92%**	**54.01%**	
Self-pay	**3.95%**	**2.36%**	
Hospital Division			<0.001
New England	**2.63%**	**4%**	
Middle Atlantic	**16.49%**	**12.2%**	
East North Central	**12.22%**	**13.87%**	
West North Central	**5.5%**	**6.28%**	
South Atlantic	**19.8%**	**19.69%**	
East South Central	**4.86%**	**6.25%**	
West South Central	**17.16%**	**14.1%**	
Mountain	**6.72%**	**7.54%**	
Pacific	**14.59%**	**16.08%**	
Hospital Size			<0.001
Small	**17.13%**	**20.16%**	
Medium	**29.02%**	**29.2%**	
Large	**53.84%**	**50.64%**	
Hosptal Teaching Status			<0.001
Rural	**5.32%**	**8.63%**	
Urban non-teaching	**13.77%**	**17.17%**	
Urban teaching	**80.89%**	**74.2%**	
Comorbidities			
Hypertension	0.08%	0.085%	0.93
Diabetes	**1.7%**	**1.11%**	<0.001
Coronary artery disease	0.05%	0.04%	0.86
Smoking	**6.57%**	**11.05%**	<0.001
Alcohol misuse	0.06%	0.09%	0.25
Drug misuse	**2.03%**	**3.07%**	<0.001
Obesity	**13.84%**	**11.17%**	<0.001
Collagen vascular disorders	0.39%	0.42%	0.71
Chronic kidney disease	0.06%	0.11%	0.18

**Table 4:** In-hospital outcomes in COVID-19-positive and negative pregnant patients in first trimester.

Variable	COVID-19-Positive First Trimester (0 to 13 Weeks)	COVID-19-Negative First Trimester (0 to 13 Weeks)	*p* Value
Disposition			0.96
Home/Routine	92.47%	92.86%	
SNF/LTAC/Nursing home	1.32%	1.08%	
Home health	2.65%	2.3%	
AMA	3.53%	3.76%	
Mechanical Ventilation	**2.15%**	**0.64%**	
	**Adjusted odds ratio ^1^** **3.31(95% CI 1.31–8.38)**		**0.01**
Mortality	0.43%	0.13%	
	Adjusted odds ratio ^1^ 5.11(95% CI 0.79–32.87)		0.08
Mean LOS (SD) in days	4.25	2.88	
	Adjusted length of stay ^1^ 1.24 day higher		0.18
Mean total charge (SD) USD	USD 39826	USD 30463	
	Adjusted total charge ^1^ USD 2254 higher		0.60
AKI	2.15%	1.82%	
	Adjusted odds ratio ^1^ 0.52(95% CI 0.15–1.75)		0.29
AKI requiring HD	0.43%	0.06%	
	Adjusted odds ratio unable to be calculated		
VTE	3.87%	2.17%	
	Adjusted odds ratio ^1^ 1.19(95% CI 0.55–2.56)		0.64
Threatened abortion	0.86%	1.25%	
	Adjusted odds ratio ^1^ 0.62(95% CI 0.15–2.5)		0.51
Missed abortion	2.58%	2.04%	
	Adjusted odds ratio ^1^ 1.19(95% CI 0.52–2.72)		0.67
Spontaneous abortion	4.31%	3.57%	
	Adjusted odds ratio ^1^ 0.98(95% CI 0.5–1.93)		0.96
Gestational HTN	1.29%	1.51%	
	Adjusted odds ratio ^1^ 0.93(95% CI 0.28–3.08)		0.91
Gestational DM	3.01%	1.88%	
	Adjusted odds ratio ^1^ 1.27(95% CI 0.56–2.87)		0.56
Molar pregnancy	0.43%	0.12%	
	Adjusted odds ratio ^1^ 1.42(95% CI 0.4–5.02)		0.58
Ectopic pregnancy	4.31%	5.92%	
	Adjusted odds ratio ^1^ 0.66(95% CI 0.34–1.3)		0.23

^1^ Adjusted for age, race, income insurance status, hospital size, teaching status and location, and co-morbidities. AKI = acute kidney injury, HD = hemodialysis, VTE = venous thromboembolism, HTN = essential hypertension, DM = diabetes mellitus, AMA = against medical advice, SNF = skilled nursing facility, LTAC = long-term acute care.

**Table 5:** In-hospital outcomes in COVID-19-positive and negative pregnant patients in second trimester.

Variable	COVID-19-Positive Second Trimester (14 to 26 Weeks)	COVID-19-Negative Second Trimester (14 to 26 Weeks)	*p* Value
Disposition			<0.001
Home/Routine	92.76%	94.25%	
SNF/LTAC/Nursing home	0.9%	0.88%	
Home health	3.61%	1.32%	
AMA	2.71%	3.56%	
Preeclampsia	**2.86%**	**6.06%**	
	**Adjusted odds ratio ^1^** **0.39(95% CI 0.24–0.61)**		<0.001
Eclampsia	-	0.17%	
	Adjusted odds ratio unable to be calculated		
HELLP	0.72%	0.84%	
	Adjusted odds ratio ^1^ 0.68(95% CI 0.27–1.71)		0.41
Preterm labor	3.43%	4.86%	
	Adjusted odds ratio ^1^ 0.79(95% CI 0.51–1.23)		0.30
Mechanical ventilation	**4.15%**	**0.55%**	
	**Adjusted odds ratio ^1^** **5.63(95% CI 3.51–9.03)**		<0.001
Mortality	0.29%	0.05%	
	Adjusted odds ratio ^1^ 4.38(95% CI 0.45–42.01)		0.20
Mean LOS (SD) in days	5.02	4.3	
	Adjusted length of stay ^1^ 0.35 day higher		0.24
Mean total charge (SD) USD	**USD 57,401**	**USD 34,736**	
	**Adjusted total charge ^1^** **USD 16213 higher**		**<0.001**
AKI	2.29%	1.02%	
	Adjusted odds ratio ^1^ 1.79(95% CI 1.04–3.09)		0.03
AKI requiring HD	0.43%	0.058%	
	Adjusted odds ratio ^1^ 4.6(95% CI 1.13–18.64)		0.03
VTE	1.14%	0.63%	
	Adjusted odds ratio ^1^ 1.6 (95% CI 0.77–3.35)		0.21
Threatened abortion	0.29%	0.38%	
	Adjusted odds ratio ^1^ 0.68(95% CI 0.15–3.03)		0.62
Missed abortion	**2%**	**5.89%**	
	**Adjusted odds ratio ^1^** **0.35(95% CI 0.2–0.6)**		**<0.001**
Spontaneous abortion	1.72%	2.64%	
	Adjusted odds ratio ^1^ 0.61(95% CI 0.33–1.12)		0.11
Gestational HTN	1.57%	2.17%	
	Adjusted odds ratio ^1^ 0.75(95% CI 0.40–1.38)		0.35
Gestational DM	6.87%	3.74%	
	Adjusted odds ratio ^1^ 1.39(95% CI 1.01–1.93)		0.04

^1^ Adjusted for age, race, income insurance status, hospital size, teaching status and location, and co-morbidities. AKI = acute kidney injury, HD = hemodialysis, VTE = venous thromboembolism, HTN = essential hypertension, DM = diabetes mellitus, AMA = against medical advice, SNF = skilled nursing facility, LTAC = long-term acute care, HELLP = hemolysis, elevated liver enzymes, and low platelets.

**Table 6:** In-hospital outcomes in COVID-19-positive and negative pregnant patients in third trimester.

Variable	COVID-19-Positive Third Trimester (27 Weeks to Birth)	COVID-19-Negative Third Trimester (27 Weeks to Birth)	*p* Value
Disposition			**<0.001**
Home/Routine	**97.88%**	**98.83%**	
SNF/LTAC/Nursing home	**0.07%**	**0.05%**	
Home health	**1.26%**	**0.81%**	
AMA	**0.78%**	**0.3%**	
Preeclampsia	**9%**	**7.05%**	
	**Adjusted odds ratio ^1^** **1.17(95% CI 1.09–1.26)**		**<0.001**
Eclampsia	0.13%	0.069%	
	Adjusted odds ratio ^1^ 1.56(95% CI 0.89–2.72)		0.11
HELLP	**0.45%**	**0.24%**	
	**Adjusted odds ratio ^1^** **1.78(95% CI 1.3–2.44)**		**<0.001**
Preterm labor	1.22%	0.76%	
	Adjusted odds ratio ^1^ 1.29(95% CI 1.05–1.58)		0.01
Mechanical ventilation	**0.84%**	**0.05%**	
	**Adjusted odds ratio ^1^** **14.8(95% CI 11.1–19.8)**		**<0.001**
Mortality	**0.093%**	**0.0039%**	
	**Adjusted odds ratio ^1^** **24.4(95% CI 10.7–55.63)**		**<0.001**
Mean LOS in days	**2.78**	**2.47**	
	**Adjusted length of stay ^1^** **0.27 day higher**		**<0.001**
Mean total charge USD	**USD 29,128**	**USD 23,039**	
	**Adjusted total charge ^1^** **USD 4454 higher**		**<0.001**
AKI	0.34%	0.15%	
	Adjusted odds ratio ^1^ 1.71(95% CI 1.18–2.47)		0.004
AKI requiring HD	**0.031%**	**0.002%**	
	**Adjusted odds ratio ^1^** **14.33(95% CI 3.69–55.69)**		**<0.001**
VTE	0.093%	0.068%	
	Adjusted odds ratio ^1^ 1.31(95% CI 0.67–2.56)		0.41
Gestational HTN	6.37%	7.4%	
	Adjusted odds ratio ^1^ 0.88(95% CI 0.81–0.96)		0.005
Gestational DM	9.84%	9.22%	
	Adjusted odds ratio ^1^ 0.98(95% CI 0.91–1.05)		0.68

^1^ Adjusted for age, race, income insurance status, hospital size, teaching status and location, and co-morbidities. AKI = acute kidney injury, HD = hemodialysis, VTE = venous thromboembolism, HTN = essential hypertension, DM = diabetes mellitus, AMA = against medical advice, SNF = skilled nursing facility, LTAC = long-term acute care, HELLP = hemolysis, elevated liver enzymes, and low platelets.

**Table 7:** Obstetric outcomes in the second trimester.

Variable	COVID-19-Positive Second Trimester (14 to 26 Weeks)	COVID-19-Negative Second Trimester (14 to 26 Weeks)	*p* Value
Vaginal delivery	54.31%	57.07%	0.53
C-section	41.38%	40.2%	0.79

**Table 8:** Obstetric outcomes in the third trimester.

Variable	COVID-19-Positive Third Trimester	COVID-19-Negative Third Trimester	*p* Value
Vaginal delivery	62.5%	63.66%	0.02
C-section	33.42%	32.07%	0.005

## Data Availability

The data used in this study are from the National (Nationwide) Inpatient Sample for the year 2020, obtained from the Healthcare Cost and Utilization Project. The NIS is a publicly available database, and researchers interested in accessing the data can obtain these directly from the HCUP website (https://www.hcup-us.ahrq.gov, accessed on 5 February 2023).

## Supplementary Materials

The following supporting information can be downloaded at: https://www.mdpi.com/article/10.3390/biomedicines11112886/s1, Table S1: ICD-10 codes. Table S2: Most common reason for admission (COVID negative 1st trimester). Table S3: Most common reason for admission (COVID negative 2nd trimester). Table S4: Most common reason for admission (COVID negative 3rd trimester).
