# Supplementary material for: Impact of COVID-19 on Pregnancy Outcomes across Trimesters in the United States
Source: Biomedicines. 2023 Oct 25;11(11):2886. doi: 10.3390/biomedicines11112886 (PMC10669814; doi:10.3390/biomedicines11112886)
Supplement: Supplementary file 1 [file biomedicines-11-02886-s001.zip › biomedicines-2615893-supplementary.pdf]

Supplementary Table S1: ICD 10 codes	
**Variable**	**ICD-10 CM code**
Covid-19	U071, U00, U49, U50, U85, J1282
AKI	N17.XX, N99.0
First Trimester	Z3A.0, Z3A.10 - Z3A.13
Second Trimester	Z3A.14 - Z3A.27
Third Trimester	Z3A.28, Z3A.29, Z3A.3, Z3A.4
Cardiac arrest	I46.XX, I49.0XX, I97.12XX, I97.71XX
Smoking	F17.XX, Z87.891
Gestational HTN	O13.XX
Gestational DM	O24.4XX
Preeclampsia	O11.XX, O14.OX, O14.1X, O14.9X
Eclampsia	O15.XX
HELLP	O14.2
Preterm labor	O60.XX
Threatened abortion	O20.0XX
Missed abortion	O02.1XX
Spontaneous abortion	O03.XX
Molar pregnancy	O01.XX
Ectopic pregnancy	O00.XX
VTE	I82.4XX, I82.6XX, I82.B1X, I82.A1X, I82.C1X, I26.XX, I82.0, I82.1, I82.210, I82.220, I82.290 I82.3X, I82.890, I82.90, I82.81X
HTN, DM, CKD, CAD, Collagen vascular disorders, Obesity, alcohol, drug abuse	Elixhauser comorbidities were used
**Variable**	**ICD-10 procedure code**
Intubation	5A1945Z, 5A1955Z, 5A1935Z, 5A09357, 5A09457, 5A09557
Vasopressor use	3E030XZ, 3E033XZ, 3E040XZ, 3E043XZ, 3E050XZ, 3E053XZ, 3E060XZ, 3E063XZ
Hemodialysis	5A1D70Z, 5A1D90Z, 5A1D80Z, 5A1D00Z, 5A1D60Z

Supplemental Table S2: Most common reason for admission (COVID negative 1st trimester)	
Diagnosis	N (%)
1. Hyperemesis gravidarum with metabolic disturbance	5530 (14.35%)
2. Mild hyperemesis gravidarum	3640 (9.44%)
3. Mental disorders complicating pregnancy	1825 (4.73%)
4. Diseases of the digestive system complicating pregnancy	1395 (3.62%)
5. Infections of kidney in pregnancy	1276 (3.36%)
6. Maternal infectious and parasitic diseases complicating pregnancy	1011 (2.62%)
7. Maternal care for low transverse scar from previous cesarean delivery	955 (2.47%)
8. Drug use complicating pregnancy	741 (1.92%
9. Injury, poisoning and certain other consequences of external causes complicating pregnancy	739 (1.91%)
10. Pre-existing type 2 diabetes mellitus, in pregnancy	701 (1.82%)

Supplemental Table S3: Most common reason for admission (COVID negative 2nd trimester)	
Diagnosis	N (%)
1. Maternal care for intrauterine death	6125 (6.45%)
2. Preterm labor	5805 (6.11%)
3. Infections of kidney in pregnancy	5630 (5.93%)
4. Missed abortion	5065 (5.33%)
5. Preterm premature rupture of membranes, unspecified as to length of time between rupture and onset of labor	4940 (5.2%)
6. Maternal care for cervical incompetence	3565 (3.75%)
7. Preterm labor without delivery	3175 (3.34%)
8. Cervical shortening	2805 (2.95%)
9. Diseases of the digestive system complicating pregnancy	2501 (2.63%)
10. Preterm premature rupture of membranes, onset of labor more than 24 hours following rupture	2306 (2.43%)

Supplemental Table S4: Most common reason for admission (COVID negative 3rd trimester)	
Diagnosis	N (%)
1. Maternal care for low transverse scar from previous cesarean	362080 (10.92%)
2. Post-term pregnancy	286115 (8.62%)
3. Streptococcus B carrier state complicating childbirth	161920 (4.88%)
4. Gestational [pregnancy-induced] hypertension without significant proteinuria, complicating childbirth	132810 (4%)
5. Abnormality in fetal heart rate and rhythm complicating labor and delivery	119080 (3.59%)
6. Second degree perineal laceration during delivery	115200 (3.47%
7. Full-term premature rupture of membranes, onset of labor within 24	105630 (3.18%)
8. First degree perineal laceration during delivery	101215 (3.05%)
9. Labor and delivery complicated by cord around neck, without compression	97035 (2.92%)
10. Encounter for full-term uncomplicated delivery	92270 (2.78%)
